# Comparative mitogenomics of *Roscoea alpina* and *R. purpurea* from contrasting elevations reveals mitochondrial genomic differentiation

**DOI:** 10.3389/fpls.2026.1967948

**Published:** 2026-10-05

**Authors:** Ya-Li Wang, Jian-Li Zhao

**Affiliations:** 1 State Key Laboratory of Vegetation Structure, Function and Construction (VegLab), Yunnan Key Laboratory of Biological Adaptation, Conservation and Utilization, and Institute of Biodiversity, School of Ecology and Environmental Science, Yunnan University, Kunming, China; 2 Institute of International Rivers and Eco-Security, Yunnan University, Kunming, China

**Keywords:** comparative mitogenomics, high-altitude adaptation, mitochondrial gene differences, mitochondrial genome, repeat-mediated recombination, Roscoea

## Abstract

Plant mitochondrial genomes are structurally dynamic and contribute to energy metabolism and stress responses. Comparative mitogenomic analyses of closely related species occupying contrasting elevations can provide insights into mitochondrial genomic differentiation across elevational environments. Using PacBio HiFi reads, we assembled and compared the mitochondrial genomes of the higher-altitude species *R. alpina* and the lower-altitude species *R. purpurea*. Both mitochondrial genome assemblies yielded graph-like structures, with *R. alpina* exhibiting a more complex assembly-graph topology, more dispersed repeats, and extensive evidence of dispersed-repeat-mediated recombination. Despite these structural differences, the two species retained broadly similar core gene complements, codon-usage patterns, and predicted RNA-editing profiles. Among genes represented by multiple annotated copies in both species, 12 had higher annotated copy numbers in *R. alpina*. Most shared protein-coding genes were highly conserved, whereas *nad4* and *rps3* each contained two nonsynonymous substitutions between the two species. Genes with higher annotated copy numbers and those showing limited sequence divergence were both annotated into respiratory complex I and mitochondrial translation. These results reveal pronounced differentiation in mitochondrial genome architecture alongside strong conservation of coding characteristics. Both the differences in gene representation and the limited sequence divergence converge on the same functional processes, suggesting a potential link between mitochondrial genomic changes and adaptation to high-altitude environments. We hypothesize that these genomic features may be relevant to the physiological performance of *R. alpina* in a high-altitude environment.

## 1 Introduction

Mitochondria are semi-autonomous organelles with essential roles in plant energy production, primary metabolism, redox regulation, and cellular signaling (Vakifahmetoglu-Norberg et al., 2017). They produce ATP through oxidative phosphorylation and interact closely with photosynthesis, photorespiration, and carbon and nitrogen metabolism. Although most mitochondrial proteins are nuclear-encoded, plant mitochondrial genomes retain genes involved in respiration, ATP synthesis, cytochrome c biogenesis, membrane transport, and mitochondrial translation (Palmer, 1979; Van Aken, 2021). Plant mitochondrial genomes are also structurally dynamic: abundant noncoding and repetitive sequences promote recombination and diverse genomic configurations, whereas coding sequences generally remain conserved (Gualberto and Newton, 2017; Palmer and Herbon, 1988; Wang et al., 2024a).

Previously, plant mitochondrial genome assembly was challenging because sequencing coverage is uneven among genomic compartments and because mitochondrial genomes contain large repeats and intracellularly transferred DNA fragments (Wang et al., 2024b). Recent advances in long-read sequencing and genome-assembly algorithms have substantially improved the reconstruction of complex genomic regions (Yang et al., 2025). For plant mitochondrial genomes, tools such as PMAT (Bi et al., 2024), HiMT (Tang et al., 2025), and Oatk (Zhou C. et al., 2025), have substantially improved the recovery and assembly of plant mitochondrial genomes from whole-genome sequencing data.

High-altitude plants experience low temperatures, reduced oxygen partial pressure, and strong ultraviolet radiation, all of which can affect energy metabolism and cellular redox balance (Zhang et al., 2024). Mitochondria may contribute to high-altitude responses by regulating ATP production, respiratory electron transport, reactive oxygen species homeostasis, and retrograde signaling (Liu et al., 2025; Punkkinen et al., 2026). Comparative mitogenomic studies across elevational gradients remain limited; a recent comparison of the alpine Corydalis impatiens and the lowland C. pauciovulata identified mitochondrial differences potentially associated with altitude (Li et al., 2026).

The genus *Roscoea* is the only predominantly alpine lineage in the family Zingiberaceae and is mainly distributed in the Himalayas and the Hengduan Mountains. *R. alpina* and *R. purpurea* are closely related Himalayan species that occupy contrasting elevations: *R. alpina* occurs mainly in high-altitude alpine meadows, whereas *R. purpurea* is usually found at lower elevations under forest canopies (Cowley, 2007; Zhao et al., 2021, 2017). Phylogenomic analyses suggest that *R. alpina* diverged from the lineage leading to *R. purpurea* approximately 14 million years ago, indicating a long history of differentiation along the elevational gradient (Zhao et al., 2021). Previous studies also revealed extensive nuclear genomic differences between the two species and adaptive signals in the plastid genes of high-altitude *Roscoea* species (Lu et al., 2025; Wang, Li, et al., 2024). Their mitochondrial genomes, however, remain poorly characterized, despite the central roles of mitochondria in respiration, energy production, and stress responses.

In this study, we compared the mitochondrial genomes of *R. alpina* and *R. purpurea* to characterize genomic differences associated with their contrasting elevational environments. Specifically, we examined genome organization, gene content, repetitive sequences, and dispersed-repeat-mediated recombination, and we compared codon usage, predicted RNA editing, and pairwise sequence divergence among shared protein-coding genes. These analyses aimed to identify mitochondrial genomic features potentially associated with the high-altitude adaptation of *R. alpina*.

## 2 Materials and methods

### 2.1 Plant material, DNA extraction, sequencing

Fresh leaves of *R. alpina* and *R. purpurea* were collected from cultivated plants maintained at Yunnan University. Total genomic DNA was extracted from fresh leaf tissues using a modified cetyltrimethylammonium bromide (CTAB) method (Aboul-Maaty and Oraby, 2019). DNA quality and integrity were assessed before library construction, and only high-quality samples were used for sequencing. PacBio High-Fidelity (HiFi) libraries were prepared using the circular consensus sequencing (CCS) strategy and sequenced on the PacBio Sequel II platform. Sequencing depth was at least 30× for each species.

### 2.2 Mitochondrial genome assembly and assembly graph complexity analysis

PacBio HiFi reads generated in BAM format were first converted to FASTQ format using the bam2fastq module in bam2fastx. The mitochondrial genomes of *R. alpina* and *R. purpurea* were then assembled *de novo* with PMAT (Bi et al., 2024), using the parameters -fc 0.5 -sd 10 -ml 100 -mi 90. Mitochondrial assembly graphs were visualized using Bandage v0.8.1 (Wick et al., 2015). For graph-based mitochondrial assemblies, assembly size was defined as the cumulative sequence span of the graph rather than the physical size of a single mitochondrial genome configuration.

To quantify mitochondrial assembly-graph complexity, the GFA files generated by PMAT were parsed and analyzed using graph-based metrics. These metrics included node and edge counts, total node length, node-length distribution, connected components, branching nodes, and node-degree distribution. Nodes with degree ≥3 were defined as branching nodes and were used to represent putative branching regions in the assembly graph.

Cyclic complexity was quantified using the cyclomatic number, calculated as E - V + C, where E is the number of edges, V is the number of nodes, and C is the number of connected components. To account for differences in graph size, node-normalized cyclic complexity was calculated by dividing the cyclomatic number by the total number of nodes. These metrics were used to compare mitochondrial assembly-graph complexity between *R. alpina* and *R. purpurea*.

### 2.3 Genome annotation

Gene annotation: Mitochondrial genomes were annotated using PMGA (Li et al., 2025). Protein-coding genes, ribosomal RNA genes, and transfer RNA genes were identified, and their coordinates, strand orientations, gene names, and feature types were recorded. Annotations were compared between species to identify differences in gene presence and annotated copy number. Differences were classified as: type I, genes annotated in only one species; type II, genes represented by one annotated copy in one species and multiple copies in the other; and type III, genes represented by multiple annotated copies in both species but differing in copy number. Genes in these categories were subsequently searched against Swiss-Prot for functional annotation.

Repeat annotation: Repetitive sequences were characterized in the mitochondrial genomes of both species. Three repeat classes were analyzed: simple sequence repeats (SSRs), tandem repeats, and dispersed repeats. SSRs were identified by scanning repeat motifs across the mitochondrial assembly sequences with custom scripts. Tandem repeats were detected using Tandem Repeats Finder (TRF) (Benson, 1999) with the parameters 2 7 7 80 10 50 500. Repeat records were summarized by class, type, copy number, length distribution, and total annotated repeat length.

### 2.4 Identification of repeat-mediated recombination

Because dispersed repeats are directly associated with homologous recombination and structural rearrangement in plant mitochondrial genomes, dispersed-repeat pairs were used as candidate recombination substrates (Kozik et al., 2019; Yu et al., 2025). Potential repeat-mediated recombination was evaluated using a modified strategy based on the Trachelospermum jasminoides mitochondrial genome, in which alternative repeat-associated conformations were assessed using long-read mapping evidence (Cai et al., 2024).

For each candidate dispersed-repeat pair, both repeat copies and 500-bp flanking sequences on each side were extracted to construct the c1 and c2 references representing the assembled conformations. Alternative recombined junctions were then generated computationally according to repeat orientation, producing the corresponding c3 and c4 reference conformations.

PacBio HiFi reads were mapped to the c1-c4 conformation references using minimap2 v2.28-r1209 (Li, 2018). Alignments were sorted, indexed, and summarized with SAMtools v1.24 (Li et al., 2009). Reads mapped to c1 and c2 were counted as support for the assembled conformations, whereas reads mapped to c3 and c4 supported the recombined conformations. Relative recombination frequency was calculated as the proportion of reads supporting c3 and c4 among all reads mapped to c1-c4.

### 2.5 Codon usage bias analysis

To assess mitochondrial codon usage, protein-coding sequences were extracted from the mitochondrial genome annotations. Codons were parsed according to the coding frame, and stop codons (UAA, UAG, and UGA) were excluded from subsequent RSCU calculations. Relative synonymous codon usage (RSCU) values were calculated for the 61 sense codons in each species. Codons with RSCU > 1 were considered preferentially used, whereas those with RSCU < 1 were considered underrepresented. Similarity between the RSCU profiles of *R. alpina* and *R. purpurea* was evaluated using Pearson’s correlation across the 61 sense codons. Codon-usage profiles were then visualized and compared between species.

### 2.6 Prediction of mitochondrial RNA editing sites

Candidate mitochondrial RNA-editing sites were predicted with Deepred-Mt (Edera et al., 2021), a deep-learning tool developed for plant mitochondrial RNA editing. Protein-coding sequences were extracted from the cleaned mitochondrial annotations and used as input. Because plant mitochondrial RNA editing predominantly involves cytidine-to-uridine conversion, only predicted C-to-U sites were retained for downstream analyses. For each predicted site, the gene name, nucleotide position, codon position, codon change, amino acid change, and editing type were recorded. Predicted sites were classified as synonymous, nonsynonymous, or stop-gained according to their effects on coding sequences. Candidate sites were summarized by gene and species and then compared between *R. alpina* and *R. purpurea*. Because the sites were computationally inferred, they were treated as candidate RNA-editing sites rather than experimentally validated events.

### 2.7 Pairwise sequence divergence analysis of mitochondrial protein-coding genes

Mitochondrial protein-coding genes shared by *R. alpina* and *R. purpurea* were identified from the cleaned genome annotations for pairwise sequence comparison. Coding sequences were checked for completeness and reading-frame integrity before alignment. For genes with complex exon structures, particularly trans-spliced *nad* genes, mature coding sequences were re-examined and reconstructed according to the annotated exon order and strand orientation in the GFF3 files.

Protein sequences of homologous gene pairs were aligned using MAFFT, and the corresponding codon alignments were generated by back-translation with PAL2NAL. Terminal stop codons were removed before subsequent analysis, and alignments were checked for internal stop codons, ambiguous bases, and frame-disrupting gaps. Pairwise nonsynonymous substitution rates (*d_N_*), synonymous substitution rates (*d_S_*), and *d_N_/d_S_* ratios were estimated using the yn00 program implemented in PAML, based on the Yang–Nielsen method (Yang, 2007). For gene pairs with *d_S_* = 0, the *d_N_/d_S_* ratio was treated as undefined and was not used for evolutionary interpretation. Sequence divergence was therefore evaluated from the estimated *d_N_* and *d_S_* values together with the numbers and types of codon differences between the two species. Genes containing nonsynonymous differences were further examined using their mitochondrial functional annotations.

## 3 Results

### 3.1 Graph-like mitochondrial genome assemblies and comparison of graph complexity

The mitochondrial genomes of *R. alpina* and *R. purpurea* were assembled as graph-like structures rather than single circular molecules (**Figures 1A, B**). Total assembled sequence lengths were 9,419,238 bp in *R. alpina* and 8,991,288 bp in *R. purpurea*. These values refer to the cumulative sequence spans represented in the mitochondrial assembly graphs. Assembly N50 values were 269,829 bp and 539,642 bp, and mean segment lengths were 81,200.33 bp and 179,825.76 bp, respectively. Mitochondrial GC content was similar between species (44.34% in *R. alpina* vs. 44.49% in *R. purpurea*).

**Figure 1:**
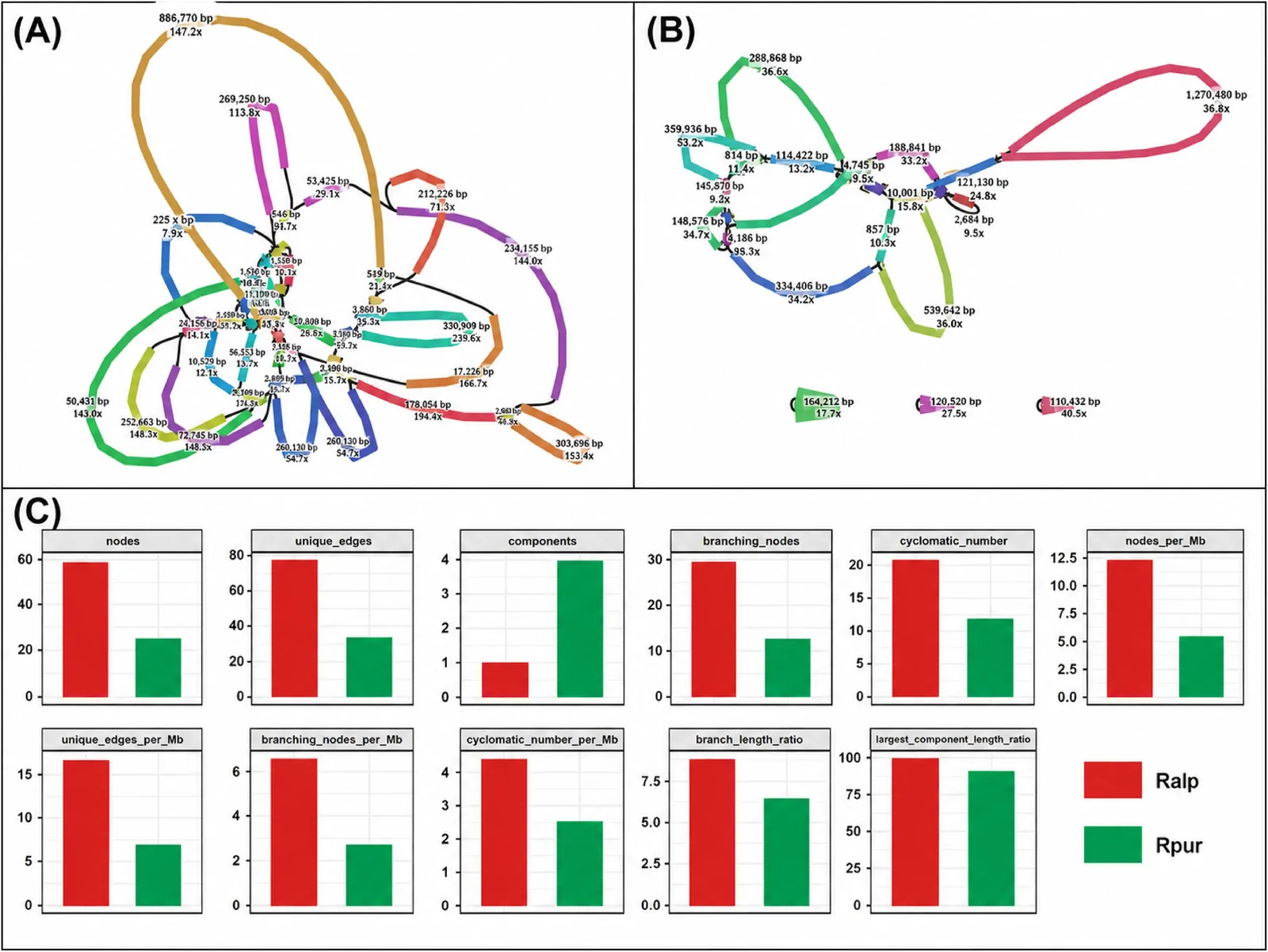
Visualization and complexity comparison of mitochondrial assembly graphs in *R. alpina* and *R. purpurea*. **(A, B)** Assembly graphs of *R. alpina* and *R. purpurea*, respectively. Colored segments represent assembled mitochondrial sequences; labels indicate segment length and coverage depth. **(C)** Comparison of assembly-graph complexity metrics between the two species.

Assembly-graph topology differed substantially between the two species (**Figure 1C**). The *R. alpina* graph contained 58 nodes and 78 unique edges within a single connected component. In contrast, the *R. purpurea* graph contained 26 nodes and 34 unique edges distributed across four connected components. *R. purpurea* also had a larger maximum segment length and higher segment N50, indicating fewer but generally longer assembled segments. The *R. alpina* assembly graph showed greater branching and cyclic complexity, with more branching nodes than *R. purpurea* (30 vs. 13), a higher maximum node degree (5 vs. 4), and a higher cyclomatic number (21 vs. 12). Length-normalized metrics showed the same general pattern, with more nodes, unique edges, branching nodes, and cycles per Mb in *R. alpina*. The branch-length ratio was also higher in *R. alpina* (8.75% vs. 6.68%), whereas tip nodes occurred only in *R. purpurea*. Together, these metrics demonstrate that the *R. alpina* assembly graph was more highly branched and topologically complex.

### 3.2 Comparative mitochondrial gene annotation

The mitochondrial gene complements and annotated copy numbers of *R. alpina* and *R. purpurea* are summarized in **Supplementary Table S1**. Annotation identified 66 distinct gene types in *R. alpina*, comprising 38 protein-coding genes, two ribosomal RNA genes, and 26 transfer RNA genes, with a total of 223 annotated gene copies. *R. purpurea* contained 65 distinct gene types, including 36 protein-coding genes, two ribosomal RNA genes, and 27 transfer RNA genes, with 200 annotated copies. Both species retained broadly similar gene complements associated with oxidative phosphorylation, cytochrome c biogenesis, ribosomal function, RNA maturation, and mitochondrial protein transport.

Comparison of the mitochondrial genome annotations identified 27 genes that differed in presence or annotated copy number. Eleven genes were classified as type I, and no type II genes were detected. Six type I genes were annotated only in *R. alpina*: *atp4*, *nad7*, *rpl2*, *rps19*, *trnI-AAU*, and *trnP-GGG*. The remaining 16 genes were classified as type III because they had multiple annotated copies in both species but differed in copy number. Of these, 12 type III genes had more annotated copies in *R. alpina*, including *nad1*, *nad4L*, *nad5*, and nine tRNA genes, whereas four genes (*ccmFN*, *nad2*, *trnK-UUU*, and *trnV-GAC*) had more annotated copies in *R. purpurea* (**Table 1**).

**Table 1:** Classification and functional annotation of mitochondrial genes showing interspecific differences in presence or annotated copy number between *R. alpina* and *R. purpurea*.

Difference type	Genes	Main functional categories
Type I: *R. alpina* only	*atp4*, *nad7*, *rpl2*, *rps19*, *trnI-AAU*, *trnP-GGG*	Oxidative phosphorylation; mitochondrial translation
Type I: *R. purpurea* only	*cox1*, *nad6*, *trnI-GAU*, *trnT-GGU*, *trnV-CAC*	Oxidative phosphorylation; mitochondrial translation
Type II	None detected	—
Type III: more copies in *R. alpina*	*nad1*, *nad4L*, *nad5*, *trnE-UUC*, *trnF-GAA*, *trnG-GCC*, *trnH-GUG*, *trnI-CAU*, *trnP-UGG*, *trnQ-UUG*, *trnS-GCU*, *trnY-GUA*	Oxidative phosphorylation; mitochondrial translation
Type III: more copies in *R. purpurea*	*ccmFN*, *nad2*, *trnK-UUU*, *trnV-GAC*	Cytochrome c biogenesis; oxidative phosphorylation; mitochondrial translation

Genes differing in representation were mainly associated with oxidative phosphorylation, mitochondrial translation, and cytochrome c biogenesis. Among the 11 protein-coding genes, eight encoded oxidative-phosphorylation components, two encoded mitochondrial ribosomal proteins, and one was associated with cytochrome c maturation. Swiss-Prot searches supported functional assignments for 10 protein-coding genes. No significant Swiss-Prot match was recovered for *nad2*, whose function was retained from the curated mitochondrial annotation. The remaining 16 genes were tRNAs involved in mitochondrial codon recognition and translation.

### 3.3 Repeat sequence composition and length distribution

Repeat annotation revealed clear differences in repeat composition between the mitochondrial genomes of *R. alpina* and *R. purpurea* (**Supplementary Table S2**). *R. alpina* contained more annotated repeat copies than *R. purpurea* (8,737 vs. 7,941), including more SSRs (3,318 vs. 3,119; **Figure 2A**), dispersed repeats (224 vs. 44; **Figure 2B**), and tandem repeats (5,195 vs. 4,778; **Figure 2C**).

**Figure 2:**
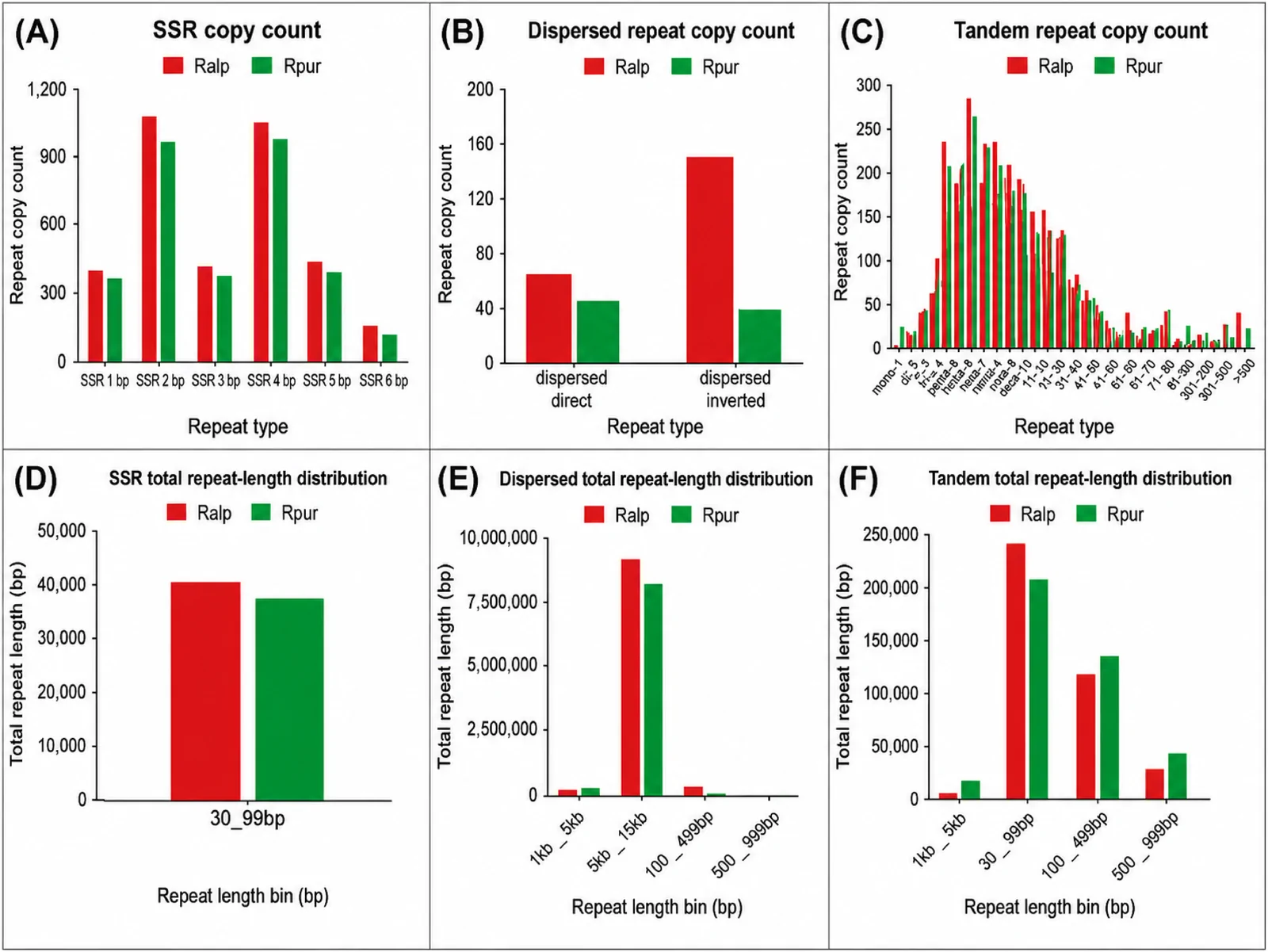
Comparative profiles of repetitive sequences in the mitochondrial genomes of *R. alpina* and *R. purpurea*. **(A)** Copy numbers of simple sequence repeats (SSRs) classified according to motif length from 1 to 6 bp. **(B)** Copy numbers of direct and inverted dispersed repeats. **(C)** Copy-number distribution of tandem repeats across different repeat periods. **(D–F)** Total sequence lengths contributed by SSRs, dispersed repeats, and tandem repeats, respectively, grouped into repeat-length intervals. Red and green bars represent *R. alpina* (Ralp) and *R. purpurea* (Rpur), respectively.

The largest interspecific difference involved dispersed repeats. *R. alpina* contained approximately five times more dispersed-repeat records than *R. purpurea*, including 68 direct and 156 inverted repeats; by contrast, all 44 dispersed repeats identified in *R. purpurea* were inverted (**Figure 2B**). Although less abundant than SSRs and tandem repeats, dispersed repeats contributed the greatest summed repeat length in both species (**Figure 2E**). *R. alpina* had a greater total dispersed-repeat length, whereas individual dispersed repeats in *R. purpurea* had larger mean and maximum lengths. Thus, *R. alpina* contained more dispersed repeats with both direct and inverted orientations, whereas *R. purpurea* contained fewer but generally longer dispersed repeats (**Figures 2B, E**).

Tandem repeats were the most abundant repeat class in both species, accounting for 59.46% of all annotated repeats in *R. alpina* and 60.17% in *R. purpurea*. Their copy-number distributions were broadly similar, although *R. alpina* had higher counts across many repeat categories (**Figure 2C**). SSR abundance showed a similar pattern, with *R. alpina* generally containing more copies across most motif classes (**Figure 2A**).

Repeat-length distributions were broadly similar between the two species. Most annotated repeat copies belonged to the 30–99 bp class, comprising 7,776 repeats in *R. alpina* and 7,120 in *R. purpurea*, equivalent to 89.00% and 89.66% of all annotated repeats, respectively (**Supplementary Table S2**). *R. alpina* contained more repeats in the 30–99 bp, 100–499 bp, and ≥ 5 kb classes, whereas *R. purpurea* contained slightly more repeats in the 500–999 bp and 1–5 kb classes. For SSRs, the 30–99 bp class contributed a greater total repeat length in *R. alpina* (**Figure 2D**). Tandem repeats were concentrated mainly in the 30–99 bp and 100–499 bp classes (**Figure 2F**).

Thus, the two mitogenomes had broadly similar repeat-length distributions but differed markedly in dispersed-repeat composition. *R. alpina* contained more dispersed repeats and a broader combination of repeat orientations, whereas *R. purpurea* contained fewer but generally longer dispersed repeats. These differences in abundance, orientation, and length were consistent with the contrasting structures of their mitochondrial assembly graphs.

### 3.4 Dispersed-repeat-mediated recombination in mitochondrial genomes

Candidate dispersed-repeat-mediated alternative conformations were evaluated using PacBio HiFi read support across the c1-c4 repeat-flanking junctions. Under the filtering criteria applied in this study, supported dispersed-repeat-mediated recombination events were retained only for *R. alpina*. No event from *R. purpurea* met the criteria for inclusion in the final results table (**Supplementary Table S3**).

In *R. alpina*, 68 dispersed-repeat pairs were retained for conformation analysis. Of these, 67 showed PacBio HiFi read support for recombined conformations, whereas one lacked informative junction-spanning reads. Among the 67 supported events, 48 were classified as strongly supported, 13 as weakly supported, and 6 as having very low support. Supported events involved both repeat orientations, including 33 direct-repeat-mediated and 34 inverted-repeat-mediated events. Repeat lengths ranged from 253 bp to 538 bp, with a mean length of approximately 301 bp, indicating that short- to medium-length dispersed repeats contributed to the detected alternative conformations.

Across all retained dispersed-repeat events in *R. alpina*, 18,457 of 51,937 junction-spanning reads supported recombined conformations, corresponding to a pooled relative recombination frequency of 35.54%. The strongest read-supported events involved 538-bp repeats, including Ralp_DISP_000032, Ralp_DISP_000031, Ralp_DISP_000033, and Ralp_DISP_000030; each had more than 2,000 recombined junction-spanning reads and a relative recombination frequency of approximately 58%. These results demonstrate that a subset of dispersed repeats in *R. alpina* is associated with HiFi-supported alternative mitochondrial conformations and may contribute to its more complex assembly graph.

### 3.5 Codon-usage analysis of protein-coding genes

Codon-usage analysis included 99 coding sequences from *R. alpina* and 94 from *R. purpurea*, comprising 22,226 and 21,849 codons, respectively. Overall RSCU profiles across the 61 sense codons were highly similar between the two species (Pearson’s r = 0.992; **Figure 3A**). Twenty-seven codons had RSCU values > 1 in *R. alpina*, compared with 28 in *R. purpurea*. All 27 preferred codons in *R. alpina* were also preferred in *R. purpurea*. In GCA (encoding alanine) was additionally preferred in *R. purpurea*. (**Figure 3A**).

**Figure 3:**
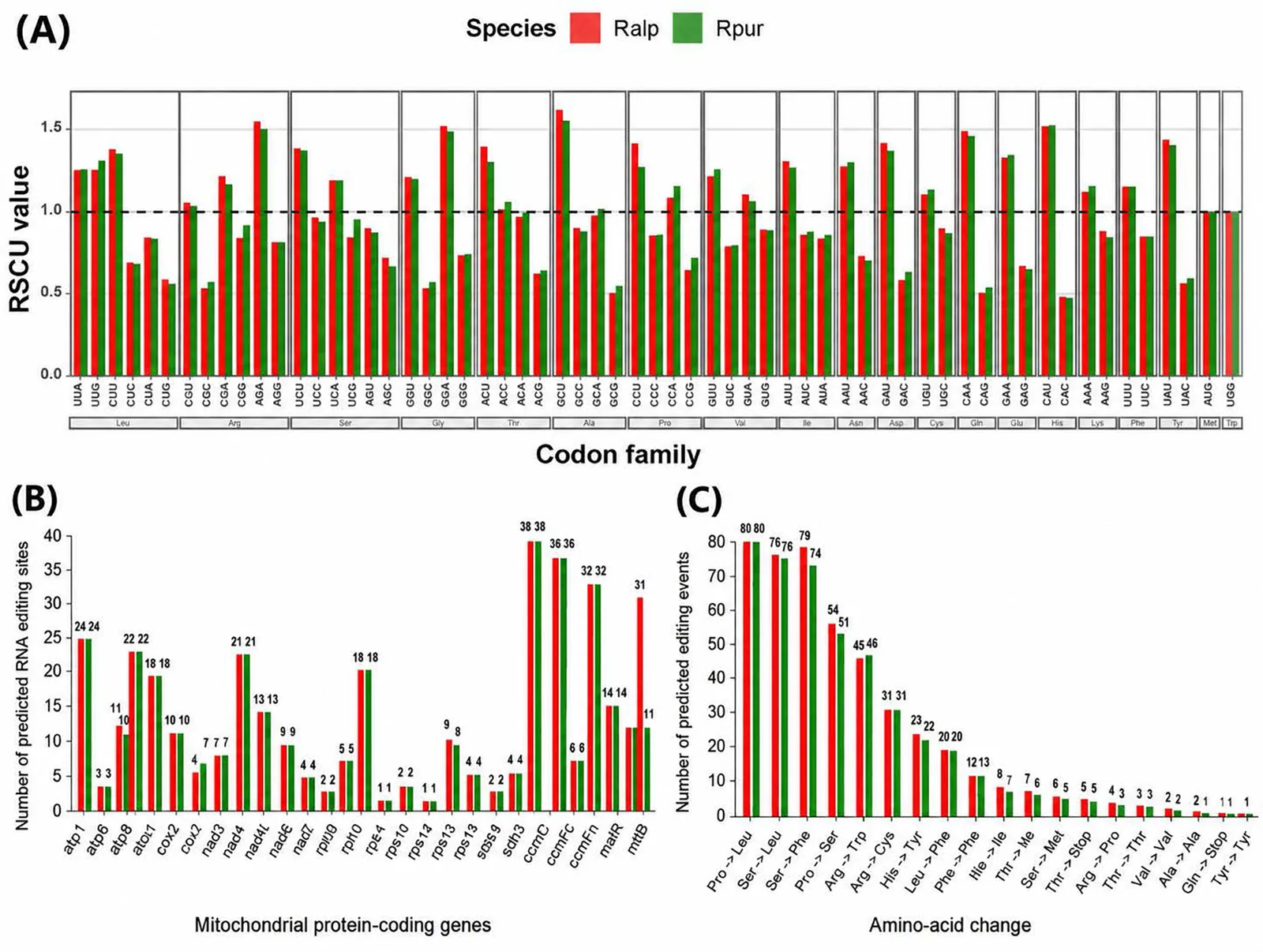
Comparative codon-usage bias and predicted RNA editing patterns in the mitochondrial genomes of *R. alpina* and *R. purpurea*. **(A)** Relative synonymous codon usage (RSCU) values for synonymous codons in mitochondrial protein-coding genes. **(B)** Numbers of predicted C-to-U RNA-editing sites in protein-coding genes shared by the two species. **(C)** Frequencies of amino acid changes resulting from the predicted RNA editing events in the shared mitochondrial protein-coding genes.

Preferred codons in both species showed a strong tendency to end in A or U. Of the preferred codons, 25 of 27 in *R. alpina* and 26 of 28 in *R. purpurea* ended in A or U; only two preferred codons in each species ended in G or C (**Figure 3A**). GCU (encoding alanine) had the highest RSCU value in both *R. alpina* (1.618) and *R. purpurea* (1.554). Other strongly preferred codons shared by both species included AGA (arginine), GGA (glycine), CAU (histidine), and CAA (glutamine). UUU and AUU were the most frequently used codons in both mitogenomes. These results indicate highly conserved codon-usage patterns with a general preference for A- or U-ending synonymous codons.

### 3.6 Prediction and comparative analysis of RNA editing sites

Deepred-Mt predicted 403 high-confidence C-to-U RNA-editing sites across 36 nonredundant mitochondrial protein-coding genes in *R. alpina* and 390 sites across 34 genes in *R. purpurea* after exclusion of *nad1* and *nad5* from the analysis (**Supplementary Table S4**). In *R. alpina*, 138 sites (34.2%) occurred at the first codon position, 235 (58.3%) at the second position, and 30 (7.4%) at the third position. The corresponding numbers in *R. purpurea* were 143 (36.7%), 219 (56.2%), and 28 (7.2%), respectively. Most predicted editing events were nonsynonymous, comprising 367 sites (91.1%) in *R. alpina* and 357 sites (91.5%) in *R. purpurea*, whereas synonymous changes accounted for 36 (8.9%) and 33 (8.5%) sites, respectively. Three predicted editing events in *R. alpina* and four in *R. purpurea* were predicted to generate stop codons.

Comparison of the 32 protein-coding genes retained for interspecific analysis identified 354 predicted editing sites in *R. alpina* and 374 in *R. purpurea* (**Supplementary Table S4**; **Figure 3B**). Twenty-seven genes (84.4%) had identical predicted editing-site counts between the two species. The highest numbers of predicted sites were observed in *ccmB*, *ccmC*, and *ccmFN*, with 38, 36, and 32 sites, respectively, in both species. Differences in predicted editing-site number were restricted to five genes: *atp9* contained 11 sites in *R. alpina* and 10 in *R. purpurea*, *mttB* contained 11 and 31 sites, *nad2* contained four and seven sites, *nad4* contained seven and six sites, and *rps3* contained nine and eight sites, respectively. The most frequent predicted amino-acid changes were Ser→Leu, Pro→Leu, Ser→Phe, Pro→Ser, and Arg→Trp. Ser→Leu occurred 72 and 74 times, Pro→Leu 71 and 75 times, Ser→Phe 47 and 50 times, Pro→Ser 41 and 45 times, and Arg→Trp 28 and 30 times in *R. alpina* and *R. purpurea*, respectively. Overall, the two species exhibited broadly similar predicted RNA-editing profiles, with gene-specific differences restricted to a small subset of the shared protein-coding genes.

### 3.7 Sequence divergence of shared mitochondrial protein-coding genes

Pairwise comparison of shared mitochondrial protein-coding genes showed a high degree of sequence conservation between *R. alpina* and *R. purpurea*. Among genes for which reliable YN00 estimates were obtained, 27 showed no synonymous or nonsynonymous differences, with both dN and dS estimated as 0. No genes contained synonymous substitutions alone. Detectable nonsynonymous sequence divergence was observed in *nad4* and *rps3*.

In *nad4*, codon 441 changed from TTC in *R. alpina* to ATC in *R. purpurea*, corresponding to a Phe-to-Ile substitution, whereas codon 464 changed from CTT to ATT, corresponding to a Leu-to-Ile substitution (**Figure 4A**). No synonymous substitutions were detected in *nad4*, and a reliable *d_N_/d_S_* ratio could not be obtained. In *rps3*, codon 214 changed from GAG to GAT, corresponding to a Glu-to-Asp substitution, and codon 340 changed from AAA to CAA, corresponding to a Lys-to-Gln substitution (**Figure 4B**). The estimated *d_N_* for *rps3* was 0.002 ± 0.0014, whereas *d_S_* was 0; consequently, the *d_N_*/*d_S_* ratio could not be reliably estimated. *nad4* was annotated as encoding NADH-ubiquinone oxidoreductase chain 4, a component of mitochondrial respiratory complex I, whereas *rps3* was annotated as the mitochondrial small ribosomal subunit protein uS3m. Thus, sequence divergence among the shared mitochondrial protein-coding genes was limited and was represented by nonsynonymous differences in *nad4* and *rps3*.

**Figure 4:**
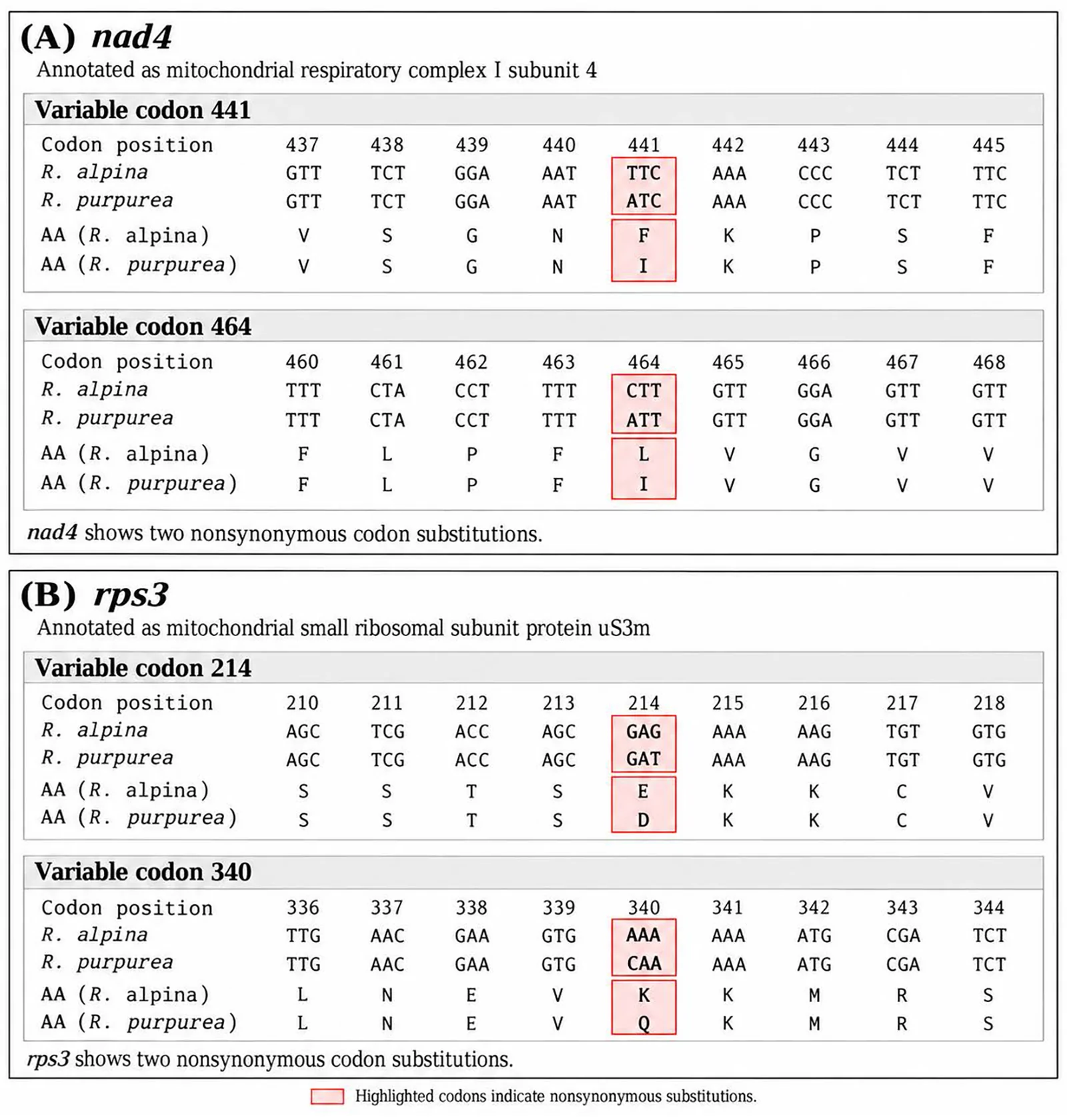
Codon-level sequence differences in the shared mitochondrial protein-coding genes *nad4* and *rps3* between *R. alpina* and *R. purpurea*. **(A)** Two nonsynonymous codon substitutions were detected in *nad4* at codon positions 441 and 464. **(B)** Two nonsynonymous codon substitutions were detected in *rps3* at codon positions 214 and 340. For each variable site, the aligned codons and the corresponding amino acids are shown for *R. alpina* and *R. purpurea*. Red boxes indicate codons and amino acids that differ between the two species.

## 4 Discussion

### 4.1 Mitochondrial genome architecture and dispersed-repeat-mediated recombination

The mitochondrial assembly graphs of *R. alpina* and *R. purpurea* differed markedly in connectivity and internal topology. The *R. alpina* assembly formed a single connected component but contained more nodes, edges, branching points, and cycles. By contrast, the *R. purpurea* assembly was divided into four connected components with fewer nodes, edges, branching nodes, and cycles. Plant mitochondrial genomes frequently contain alternative structural configurations, and graph-based representations can retain multiple possible connections without forcing the genome into a single circular model (Kozik et al., 2019; He et al., 2023). The four connected components in *R. purpurea* reflect a more partitioned assembly graph, whereas the greater branching and cyclicity of the single *R. alpina* component indicate a more complex graph topology.

This more complex graph topology in *R. alpina* coincided with a substantially higher abundance of dispersed repeats and extensive HiFi-read support for repeat-associated alternative conformations. Dispersed repeats are well-established substrates for plant mitochondrial recombination, and their abundance, length, and sequence similarity can influence DNA exchange and the formation of alternative mitochondrial configurations (Arrieta-Montiel et al., 2009; Cai et al., 2024; Liu et al., 2024). Notably, recombination-supported dispersed repeats in *R. alpina* ranged from 253 to 538 bp, closely overlapping the 108–556 bp range of recombinationally active intermediate-sized repeats reported in *Arabidopsis* (Arrieta-Montiel et al., 2009). Recent studies have likewise shown that repeats of several hundred base pairs can mediate mitochondrial recombination and generate alternative genome conformations in plants (Zhou G. et al., 2025; Xiao et al., 2026). The greater abundance of dispersed repeats in *R. alpina* may therefore provide a larger pool of potential recombination substrates and increase opportunities for alternative mitochondrial configurations.

Mitochondrial recombination and genome restructuring have been linked to plant stress responses, including thermotolerance and salt-stress tolerance, suggesting that regulated mitochondrial genome dynamics can contribute to environmental stress tolerance (Negroni et al., 2024; Shedge et al., 2010). This greater mitochondrial structural plasticity may therefore be relevant to environmental stress responses and represents a candidate feature potentially involved in responses to high-altitude conditions.

### 4.2 Comparative mitochondrial gene representation and gene annotation differences

Despite their contrasting genome architectures, *R. alpina* and *R. purpurea* retained broadly similar core mitochondrial gene complements, with major differences involving gene presence and annotated copy number. Similar conservation of mitochondrial gene content despite substantial structural and repeat variation has been reported in Digitalideae (Mower et al., 2026). Such variation may be linked to mitochondrial genome dynamics. In plants, repeat-mediated recombination can alter the relative stoichiometry of mitochondrial genomic regions through substoichiometric shifting, thereby modifying gene copy number and transcription (Arrieta-Montiel et al., 2009; Shedge et al., 2007). Mitochondrial genome copy number has also been positively associated with mitochondrial gene expression in *Arabidopsis* (Ayabe et al., 2023). The greater mitochondrial structural dynamics of *R. alpina* may therefore contribute to the higher representation of specific functional genes in its genome.

Most genes with additional annotated copies in *R. alpina* belonged to two functional groups: the respiratory complex I genes *nad1*, *nad4L*, and *nad5*, and nine mitochondrial tRNA genes. Complex I is a major entry point for electrons into the respiratory chain and is central to cellular redox and energy metabolism. Its function is particularly relevant under environmental stress: complex I defects impair cold acclimation in *Arabidopsis*, and oxygen availability alters complex I organization and activity in plant mitochondria (Lee et al., 2002; Ramírez-Aguilar et al., 2011). Similar mitochondrial signatures have been reported in the high-altitude plant *Hippophae tibetana*, where *nad4L* and *nad7* showed adaptive evolutionary signals and a mitochondrial tRNA gene occurred at high copy number (Zeng et al., 2024). Mitochondrial tRNAs are essential for organellar protein synthesis, and disruption of mitochondrial tRNA supply affects mitochondrial gene expression and plant growth (Murcha et al., 2016). The increased representation of complex I and tRNA genes in *R. alpina* may therefore have functional implications for respiration and mitochondrial translation, processes that are particularly relevant under the low-temperature and reduced-oxygen conditions characteristic of high-altitude environments.

### 4.3 Conserved codon usage and variation in predicted RNA editing

The highly similar RSCU profiles of *R. alpina* and *R. purpurea* indicate that mitochondrial codon usage remained strongly conserved during their divergence. Both species preferred A- or U-ending synonymous codons, suggesting similar nucleotide-composition and translational constraints. This conservation is consistent with the generally slow evolution of plant mitochondrial coding sequences (Palmer and Herbon, 1988) and indicates that the contrasting elevational distributions of the two species were not accompanied by broad differences in mitochondrial codon-usage patterns.

Predicted RNA editing was also broadly conserved between species. Most predicted sites occurred at the first or second codon position and caused nonsynonymous changes, a pattern widely reported in plant mitochondrial transcripts (Giegé and Brennicke, 1999). Gene-specific differences in predicted editing-site abundance were detected in a limited subset of genes, including *mttB*. Because these predictions were derived computationally from mitochondrial coding sequences, their biological significance cannot be determined from the present data and requires experimental validation.

### 4.4 Sequence divergence of mitochondrial protein-coding genes

Most mitochondrial protein-coding genes shared by *R. alpina* and *R. purpurea* were highly conserved, whereas sequence variation was concentrated in a small subset of genes (Yang et al., 2022; Zhang et al., 2022). Sequence divergence was limited to *nad4* and *rps3*, each of which contained two nonsynonymous substitutions between the two species. These substitutions indicate limited interspecific coding-sequence divergence but should not be interpreted as evidence of positive selection. *nad4* is annotated as encoding NADH-ubiquinone oxidoreductase chain 4, a component of mitochondrial respiratory complex I, whereas *rps3* is annotated as the mitochondrial small ribosomal subunit protein uS3m. Complex I plays a central role in mitochondrial respiration and energy metabolism (Kühn et al., 2015; Lee et al., 2002), whereas *rps3* is associated with mitochondrial translation (Manavski et al., 2012). Previous studies have also reported sequence variation in mitochondrial *nad* genes in high-altitude plants (Zeng et al., 2024), providing comparative context for the divergence observed in *nad4* here.

Notably, genes differing in presence or annotated copy number were also concentrated in categories associated with oxidative phosphorylation and mitochondrial translation. Together, differences in gene representation and limited protein-coding sequence divergence represent two distinct levels of mitochondrial genomic differentiation between *R. alpina* and *R. purpurea*. These patterns raise the possibility that mitochondrial genomic variation may be associated with their contrasting elevational distributions.

## 5 Conclusion

This study reveals marked mitochondrial genomic differentiation between *R. alpina* and *R. purpurea* despite broad conservation of their core coding features. *R. alpina* exhibited a more complex mitochondrial assembly graph, a greater abundance of dispersed repeats, and extensive evidence of dispersed-repeat-mediated alternative conformations, indicating greater mitochondrial structural plasticity. The two species also differed in gene presence and annotated copy number, whereas codon usage and predicted RNA-editing patterns remained largely conserved. Sequence comparisons further showed that most shared mitochondrial protein-coding genes were highly conserved, with nonsynonymous sequence divergence detected in *nad4* and *rps3*, each of which contained two nonsynonymous substitutions. Notably, differences in gene representation and protein-coding sequence divergence represent two distinct levels of mitochondrial genomic variation between the two species. Some of the genes showing variation at these levels were annotated in categories related to oxidative phosphorylation and mitochondrial translation. We hypothesize that high-altitude adaptation in *R. alpina* may be associated with mitochondrial genomic changes at these two distinct levels, which involve genes annotated in overlapping functional categories.

Comparisons among multiple closely related *Roscoea* species occupying high- and low-elevation habitats could help determine whether these mitochondrial genomic patterns are consistently associated with elevation rather than reflecting species-specific divergence. Transcriptomic and proteomic analyses under controlled environmental conditions could further evaluate the functional significance of these candidate mitochondrial features.

## Data Availability

The complete mitochondrial genome assemblies and corresponding annotation data generated in this study have been deposited in thePlant Molecular Genomics Database, accessions *Roscoea alpina*: PMGDU000064.1–PMGDU000064.58, *Roscoea purpurea*: PMGDU000063.1–PMGDU000063.25.
