# Supplementary material for: Comparative mitogenomics of Roscoea alpina and R. purpurea from contrasting elevations reveals mitochondrial genomic differentiation
Source: Front Plant Sci. 2026 Oct 5;17:1967948. doi: 10.3389/fpls.2026.1967948 (PMC13677838; doi:10.3389/fpls.2026.1967948)
Supplement: Supplementary file 1 [file Table1.docx]

Supplementary Material

# 1 Supplementary Tables

**Talbe S1. Summary of mitochondrial gene annotations and annotated copy numbers in *Roscoea alpina* and *R. purpurea*.**

| Gene category | *R. alpina* annotated genes | *R. purpurea* annotated genes |
| --- | --- | --- |
| ATP synthase | *atp1* (×2), *atp4* (×2), *atp6* (×2), *atp8* (×4), *atp9* (×6) | *atp1* (×2), *atp6* (×2), *atp8* (×4), *atp9* (×6) |
| NADH dehydrogenase | *nad1* (×2), *nad2*, *nad3* (×4), *nad4* (×2), *nad4L* (×4), *nad5* (×2), *nad7* (×2), *nad9* (×2) | *nad1*, *nad2* (×2), *nad3* (×4), *nad4* (×2), *nad4L* (×2), *nad5*, *nad6* (×2), *nad9* (×2) |
| Cytochrome b | *cob* (×2) | *cob* (×2) |
| Cytochrome c biogenesis | *ccmB* (×2), *ccmC* (×2), *ccmFC* (×2), *ccmFN* (×4) | *ccmB* (×2), *ccmC* (×2), *ccmFC* (×2), *ccmFN* (×6) |
| Cytochrome c oxidase | *cox2* (×2), *cox3* (×4) | *cox1* (×2), *cox2* (×2), *cox3* (×4) |
| Maturase | *matR* (×2) | *matR* (×2) |
| Protein transport subunit | *mttB* (×2) | *mttB* (×2) |
| Large ribosomal subunit proteins | *rpl2* (×2), *rpl5* (×2), *rpl16* (×2) | *rpl5* (×2), *rpl16* (×2) |
| Small ribosomal subunit proteins | *rps1* (×2), *rps2* (×2), *rps3* (×2), *rps4* (×2), *rps7* (×4), *rps10* (×4), *rps11* (×2), *rps12* (×2), *rps13* (×2), *rps14* (×2), *rps19* (×2) | *rps1* (×2), *rps2* (×2), *rps3* (×2), *rps4* (×2), *rps7* (×4), *rps10* (×4), *rps11* (×2), *rps12* (×2), *rps13* (×2), *rps14* (×2) |
| Succinate dehydrogenase | *sdh3* (×4), *sdh4* (×6) | *sdh3* (×4), *sdh4* (×6) |
| Ribosomal RNA | *rrn5* (×2), *rrn18* (×2) | *rrn5* (×2), *rrn18* (×2) |
| Transfer RNA | *trnA-UGC* (×2), *trnC-GCA* (×6), *trnD-GUC* (×2), *trnE-UUC* (×8), *trnF-GAA* (×12), *trnG-GCC* (×4), *trnG-UCC* (×2), *trnH-GUG* (×10), *trnI-AAU* (×2), *trnI-CAU* (×6), *trnK-UUU* (×4), *trnL-CAA* (×2), *trnL-UAG* (×2), *trnM-CAU* (×2), *trnN-GUU* (×6), *trnP-GGG* (×2), *trnP-UGG* (×8), *trnQ-UUG* (×8), *trnR-ACG* (×2), *trnR-CCG* (×2), *trnR-CCU* (×2), *trnS-GCU* (×6), *trnS-UGA* (×6), *trnV-GAC* (×6), *trnW-CCA* (×4), *trnY-GUA* (×4) | *trnA-UGC* (×2), *trnC-GCA* (×6), *trnD-GUC* (×2), *trnE-UUC* (×4), *trnF-GAA* (×8), *trnG-GCC* (×2), *trnG-UCC* (×2), *trnH-GUG* (×6), *trnI-CAU* (×4), *trnI-GAU* (×2), *trnK-UUU* (×6), *trnL-CAA* (×2), *trnL-UAG* (×2), *trnM-CAU* (×2), *trnN-GUU* (×6), *trnP-UGG* (×6), *trnQ-UUG* (×6), *trnR-ACG* (×2), *trnR-CCG* (×2), *trnR-CCU* (×2), *trnS-GCU* (×4), *trnS-UGA* (×6), *trnT-GGU* (×2), *trnV-CAC* (×2), *trnV-GAC* (×8), *trnW-CCA* (×4), *trnY-GUA* (×2) |
| Total | 38 protein-coding gene, 2 rRNA and 26 tRNA gene types | 36 protein-coding gene, 2 rRNA and 27 tRNA gene types |

**Table S2. Overall repeat class-level summary for the mitochondrial genomes of *R. alpina* and *R. purpurea*.**

| Sample | Repeat class | Repeat copy count | Copy count percentage (%) | Total annotated repeat length (bp) | Length percentage (%) | Mean repeat length (bp) | Maximum repeat length (bp) |
| --- | --- | --- | --- | --- | --- | --- | --- |
| Ralp | SSR | 3318 | 37.98 | 40851 | 0.41 | 12.31 | 35 |
| Ralp | Tandem repeats | 5195 | 59.46 | 387281 | 3.92 | 74.55 | 1221 |
| Ralp | Dispersed repeats | 224 | 2.56 | 9458712 | 95.67 | 42226.39 | 626892 |
| Ralp | All repeats | 8737 | 100 | 9886844 | 100 | 1131.61 | 626892 |
| Rpur | SSR | 3119 | 39.28 | 38670 | 0.41 | 12.4 | 60 |
| Rpur | Tandem repeats | 4778 | 60.17 | 402165 | 4.26 | 84.17 | 1799 |
| Rpur | Dispersed repeats | 44 | 0.55 | 8990640 | 95.33 | 204332.73 | 1270469 |
| Rpur | All repeats | 7941 | 100 | 9431475 | 100 | 1187.69 | 1270469 |

**Table S3.** Complete list of DRS-mediated recombination-supported events in the mitochondrial genome of *R.alpina*. c1 and c2 represent assembled conformations, whereas c3 and c4 represent putative recombined conformations; RF was calculated as the percentage of junction-spanning reads supporting c3 and c4 among all junction-spanning reads supporting c1-c4. Under the current DRS-based analytical framework, no Roscoea purpurea event met the criteria for DRS-mediated recombination support.

| **No.** | **DRS ID** | **Repeat orientation** | **Strand** | **Repeat length (bp)** | **Copy 1 position** | **Copy 2 position** | **c1** | **c2** | **c3** | **c4** | **c1+c2** | **c3+c4** | **Total** | **RF (%)** | **Support level** | **Identity** |
| --- | --- | --- | --- | --- | --- | --- | --- | --- | --- | --- | --- | --- | --- | --- | --- | --- |
| 1 | Ralp_DISP_000033 | Inverted | - | 538 | NODE_4302-_length_564_cov_985.1:1-538 | NODE_917-_length_266619_cov_153.8:242032-242569 | 199 | 1330 | 181 | 1955 | 1529 | 2136 | 3665 | 58.28 | Strong | 1.000 |
| 2 | Ralp_DISP_000032 | Direct | + | 538 | NODE_4302-_length_564_cov_985.1:1-538 | NODE_917+_length_266619_cov_153.8:24051-24588 | 190 | 1313 | 170 | 1952 | 1503 | 2122 | 3625 | 58.54 | Strong | 1.000 |
| 3 | Ralp_DISP_000030 | Inverted | - | 538 | NODE_4302+_length_564_cov_985.1:27-564 | NODE_917+_length_266619_cov_153.8:24051-24588 | 196 | 1314 | 1937 | 154 | 1510 | 2091 | 3601 | 58.07 | Strong | 1.000 |
| 4 | Ralp_DISP_000031 | Direct | + | 538 | NODE_4302+_length_564_cov_985.1:27-564 | NODE_917-_length_266619_cov_153.8:242032-242569 | 175 | 1299 | 1926 | 149 | 1474 | 2075 | 3549 | 58.47 | Strong | 1.000 |
| 5 | Ralp_DISP_000073 | Direct | + | 259 | NODE_4369-_length_289_cov_360.4:6-264 | NODE_905_1930_1959_1285+_length_332099_cov_149.717:164034-164292 | 2 | 943 | 6 | 615 | 945 | 621 | 1566 | 39.66 | Strong | 1.000 |
| 6 | Ralp_DISP_000066 | Direct | + | 259 | NODE_4369+_length_289_cov_360.4:26-284 | NODE_905_1930_1959_1285-_length_332099_cov_149.717:167808-168066 | 0 | 932 | 608 | 5 | 932 | 613 | 1545 | 39.68 | Strong | 1.000 |
| 7 | Ralp_DISP_000072 | Inverted | - | 259 | NODE_4369-_length_289_cov_360.4:6-264 | NODE_905_1930_1959_1285-_length_332099_cov_149.717:167808-168066 | 1 | 936 | 5 | 604 | 937 | 609 | 1546 | 39.39 | Strong | 1.000 |
| 8 | Ralp_DISP_000067 | Inverted | - | 259 | NODE_4369+_length_289_cov_360.4:26-284 | NODE_905_1930_1959_1285+_length_332099_cov_149.717:164034-164292 | 0 | 958 | 590 | 4 | 958 | 594 | 1552 | 38.27 | Strong | 1.000 |
| 9 | Ralp_DISP_000078 | Inverted | - | 280 | NODE_4466_4414_4515+_length_282_cov_577.31:3-282 | NODE_943-_length_212226_cov_143.1:89823-90102 | 0 | 632 | 462 | 9 | 632 | 471 | 1103 | 42.70 | Strong | 1.000 |
| 10 | Ralp_DISP_000077 | Direct | + | 280 | NODE_4466_4414_4515+_length_282_cov_577.31:3-282 | NODE_943+_length_212226_cov_143.1:122125-122404 | 0 | 565 | 451 | 11 | 565 | 462 | 1027 | 44.99 | Strong | 1.000 |
| 11 | Ralp_DISP_000081 | Inverted | - | 270 | NODE_4466_4414_4515+_length_282_cov_577.31:13-282 | NODE_986-_length_176034_cov_135.4:9761-10030 | 0 | 571 | 457 | 3 | 571 | 460 | 1031 | 44.62 | Strong | 1.000 |
| 12 | Ralp_DISP_000091 | Inverted | - | 280 | NODE_4466_4414_4515-_length_282_cov_577.31:1-280 | NODE_943+_length_212226_cov_143.1:122125-122404 | 0 | 625 | 12 | 443 | 625 | 455 | 1080 | 42.13 | Strong | 1.000 |
| 13 | Ralp_DISP_000082 | Direct | + | 270 | NODE_4466_4414_4515+_length_282_cov_577.31:13-282 | NODE_986+_length_176034_cov_135.4:166005-166274 | 0 | 528 | 437 | 2 | 528 | 439 | 967 | 45.40 | Strong | 1.000 |
| 14 | Ralp_DISP_000094 | Direct | + | 280 | NODE_4466_4414_4515-_length_282_cov_577.31:1-280 | NODE_943-_length_212226_cov_143.1:89823-90102 | 0 | 581 | 9 | 428 | 581 | 437 | 1018 | 42.93 | Strong | 1.000 |
| 15 | Ralp_DISP_000095 | Direct | + | 270 | NODE_4466_4414_4515-_length_282_cov_577.31:1-270 | NODE_986-_length_176034_cov_135.4:9761-10030 | 0 | 596 | 3 | 428 | 596 | 431 | 1027 | 41.97 | Strong | 1.000 |
| 16 | Ralp_DISP_000096 | Inverted | - | 270 | NODE_4466_4414_4515-_length_282_cov_577.31:1-270 | NODE_986+_length_176034_cov_135.4:166005-166274 | 0 | 570 | 2 | 428 | 570 | 430 | 1000 | 43.00 | Strong | 1.000 |
| 17 | Ralp_DISP_000046 | Direct | + | 302 | NODE_4363+_length_304_cov_267.5:3-304 | NODE_917-_length_266619_cov_153.8:173462-173763 | 0 | 593 | 201 | 171 | 593 | 372 | 965 | 38.55 | Strong | 0.997 |
| 18 | Ralp_DISP_000047 | Inverted | - | 302 | NODE_4363+_length_304_cov_267.5:3-304 | NODE_917+_length_266619_cov_153.8:92857-93158 | 0 | 624 | 179 | 144 | 624 | 323 | 947 | 34.11 | Strong | 0.997 |
| 19 | Ralp_DISP_000097 | Direct | + | 282 | NODE_4466_4414_4515-_length_282_cov_577.31:1-282 | NODE_917-_length_266619_cov_153.8:173511-173792 | 0 | 472 | 13 | 273 | 472 | 286 | 758 | 37.73 | Strong | 0.996 |
| 20 | Ralp_DISP_000086 | Direct | + | 280 | NODE_4466_4414_4515+_length_282_cov_577.31:3-282 | NODE_941+_length_217096_cov_148.4:39752-40031 | 0 | 245 | 275 | 3 | 245 | 278 | 523 | 53.15 | Strong | 0.996 |
| 21 | Ralp_DISP_000098 | Inverted | - | 282 | NODE_4466_4414_4515-_length_282_cov_577.31:1-282 | NODE_917+_length_266619_cov_153.8:92828-93109 | 0 | 436 | 12 | 266 | 436 | 278 | 714 | 38.94 | Strong | 0.996 |
| 22 | Ralp_DISP_000085 | Inverted | - | 280 | NODE_4466_4414_4515+_length_282_cov_577.31:3-282 | NODE_941-_length_217096_cov_148.4:177066-177345 | 0 | 239 | 272 | 3 | 239 | 275 | 514 | 53.50 | Strong | 0.996 |
| 23 | Ralp_DISP_000057 | Direct | + | 304 | NODE_4363-_length_304_cov_267.5:1-304 | NODE_941+_length_217096_cov_148.4:39779-40082 | 0 | 347 | 234 | 0 | 347 | 234 | 581 | 40.28 | Strong | 0.997 |
| 24 | Ralp_DISP_000056 | Inverted | - | 304 | NODE_4363-_length_304_cov_267.5:1-304 | NODE_941-_length_217096_cov_148.4:177015-177318 | 0 | 355 | 228 | 0 | 355 | 228 | 583 | 39.11 | Strong | 0.997 |
| 25 | Ralp_DISP_000058 | Inverted | - | 302 | NODE_4363-_length_304_cov_267.5:1-302 | NODE_917-_length_266619_cov_153.8:173462-173763 | 0 | 722 | 24 | 154 | 722 | 178 | 900 | 19.78 | Strong | 0.997 |
| 26 | Ralp_DISP_000059 | Direct | + | 302 | NODE_4363-_length_304_cov_267.5:1-302 | NODE_917+_length_266619_cov_153.8:92857-93158 | 0 | 737 | 21 | 141 | 737 | 162 | 899 | 18.02 | Strong | 0.997 |
| 27 | Ralp_DISP_000069 | Direct | + | 289 | NODE_4369-_length_289_cov_360.4:1-289 | NODE_917+_length_266619_cov_153.8:23762-24050 | 0 | 485 | 41 | 73 | 485 | 114 | 599 | 19.03 | Strong | 1.000 |
| 28 | Ralp_DISP_000063 | Direct | + | 289 | NODE_4369+_length_289_cov_360.4:1-289 | NODE_917-_length_266619_cov_153.8:242570-242858 | 0 | 471 | 79 | 31 | 471 | 110 | 581 | 18.93 | Strong | 1.000 |
| 29 | Ralp_DISP_000062 | Inverted | - | 289 | NODE_4369+_length_289_cov_360.4:1-289 | NODE_917+_length_266619_cov_153.8:23762-24050 | 0 | 504 | 84 | 26 | 504 | 110 | 614 | 17.92 | Strong | 1.000 |
| 30 | Ralp_DISP_000068 | Inverted | - | 289 | NODE_4369-_length_289_cov_360.4:1-289 | NODE_917-_length_266619_cov_153.8:242570-242858 | 0 | 486 | 20 | 81 | 486 | 101 | 587 | 17.21 | Strong | 1.000 |
| 31 | Ralp_DISP_000099 | Direct | + | 280 | NODE_4466_4414_4515-_length_282_cov_577.31:1-280 | NODE_941-_length_217096_cov_148.4:177066-177345 | 0 | 239 | 2 | 68 | 239 | 70 | 309 | 22.65 | Strong | 0.996 |
| 32 | Ralp_DISP_000038 | Inverted | - | 302 | NODE_4363+_length_304_cov_267.5:3-304 | NODE_986+_length_176034_cov_135.4:166022-166323 | 0 | 673 | 1 | 64 | 673 | 65 | 738 | 8.81 | Strong | 1.000 |
| 33 | Ralp_DISP_000051 | Inverted | - | 302 | NODE_4363-_length_304_cov_267.5:1-302 | NODE_943-_length_212226_cov_143.1:89774-90075 | 0 | 699 | 63 | 1 | 699 | 64 | 763 | 8.39 | Strong | 1.000 |
| 34 | Ralp_DISP_000052 | Direct | + | 302 | NODE_4363-_length_304_cov_267.5:1-302 | NODE_943+_length_212226_cov_143.1:122152-122453 | 0 | 713 | 64 | 0 | 713 | 64 | 777 | 8.24 | Strong | 1.000 |
| 35 | Ralp_DISP_000100 | Inverted | - | 280 | NODE_4466_4414_4515-_length_282_cov_577.31:1-280 | NODE_941+_length_217096_cov_148.4:39752-40031 | 0 | 232 | 2 | 60 | 232 | 62 | 294 | 21.09 | Strong | 0.996 |
| 36 | Ralp_DISP_000053 | Inverted | - | 302 | NODE_4363-_length_304_cov_267.5:1-302 | NODE_986-_length_176034_cov_135.4:9712-10013 | 0 | 711 | 54 | 2 | 711 | 56 | 767 | 7.30 | Strong | 1.000 |
| 37 | Ralp_DISP_000036 | Direct | + | 302 | NODE_4363+_length_304_cov_267.5:3-304 | NODE_943-_length_212226_cov_143.1:89774-90075 | 0 | 711 | 0 | 55 | 711 | 55 | 766 | 7.18 | Strong | 1.000 |
| 38 | Ralp_DISP_000039 | Inverted | - | 302 | NODE_4363+_length_304_cov_267.5:3-304 | NODE_943+_length_212226_cov_143.1:122152-122453 | 0 | 720 | 2 | 52 | 720 | 54 | 774 | 6.98 | Strong | 1.000 |
| 39 | Ralp_DISP_000044 | Direct | + | 304 | NODE_4363+_length_304_cov_267.5:1-304 | NODE_941-_length_217096_cov_148.4:177015-177318 | 0 | 354 | 0 | 52 | 354 | 52 | 406 | 12.81 | Strong | 0.997 |
| 40 | Ralp_DISP_000055 | Direct | + | 302 | NODE_4363-_length_304_cov_267.5:1-302 | NODE_986+_length_176034_cov_135.4:166022-166323 | 0 | 711 | 49 | 2 | 711 | 51 | 762 | 6.69 | Strong | 1.000 |
| 41 | Ralp_DISP_000040 | Direct | + | 302 | NODE_4363+_length_304_cov_267.5:3-304 | NODE_986-_length_176034_cov_135.4:9712-10013 | 0 | 685 | 1 | 49 | 685 | 50 | 735 | 6.80 | Strong | 1.000 |
| 42 | Ralp_DISP_000045 | Inverted | - | 304 | NODE_4363+_length_304_cov_267.5:1-304 | NODE_941+_length_217096_cov_148.4:39779-40082 | 0 | 342 | 1 | 43 | 342 | 44 | 386 | 11.40 | Strong | 0.997 |
| 43 | Ralp_DISP_000084 | Direct | + | 282 | NODE_4466_4414_4515+_length_282_cov_577.31:1-282 | NODE_917+_length_266619_cov_153.8:92828-93109 | 0 | 455 | 31 | 11 | 455 | 42 | 497 | 8.45 | Strong | 0.996 |
| 44 | Ralp_DISP_000070 | Direct | + | 281 | NODE_4369-_length_289_cov_360.4:9-289 | NODE_700-_length_626892_cov_153.9:84347-84627 | 0 | 340 | 30 | 6 | 340 | 36 | 376 | 9.57 | Strong | 1.000 |
| 45 | Ralp_DISP_000083 | Inverted | - | 282 | NODE_4466_4414_4515+_length_282_cov_577.31:1-282 | NODE_917-_length_266619_cov_153.8:173511-173792 | 0 | 464 | 27 | 9 | 464 | 36 | 500 | 7.20 | Strong | 0.996 |
| 46 | Ralp_DISP_000071 | Inverted | - | 281 | NODE_4369-_length_289_cov_360.4:9-289 | NODE_700+_length_626892_cov_153.9:542266-542546 | 0 | 338 | 27 | 4 | 338 | 31 | 369 | 8.40 | Strong | 1.000 |
| 47 | Ralp_DISP_000064 | Inverted | - | 281 | NODE_4369+_length_289_cov_360.4:1-281 | NODE_700-_length_626892_cov_153.9:84347-84627 | 1 | 337 | 4 | 26 | 338 | 30 | 368 | 8.15 | Strong | 1.000 |
| 48 | Ralp_DISP_000065 | Direct | + | 281 | NODE_4369+_length_289_cov_360.4:1-281 | NODE_700+_length_626892_cov_153.9:542266-542546 | 0 | 338 | 4 | 26 | 338 | 30 | 368 | 8.15 | Strong | 1.000 |
| 49 | Ralp_DISP_000054 | Direct | + | 302 | NODE_4363-_length_304_cov_267.5:1-302 | NODE_1052_1959_copy_975_4397_4539-_length_303065_cov_137.417:262317-262618 | 0 | 232 | 7 | 2 | 232 | 9 | 241 | 3.73 | Weak | 1.000 |
| 50 | Ralp_DISP_000042 | Inverted | - | 302 | NODE_4363+_length_304_cov_267.5:3-304 | NODE_917-_length_266619_cov_153.8:26530-26831 | 0 | 287 | 1 | 8 | 287 | 9 | 296 | 3.04 | Weak | 1.000 |
| 51 | Ralp_DISP_000041 | Direct | + | 302 | NODE_4363+_length_304_cov_267.5:3-304 | NODE_917+_length_266619_cov_153.8:239789-240090 | 0 | 281 | 0 | 7 | 281 | 7 | 288 | 2.43 | Weak | 1.000 |
| 52 | Ralp_DISP_000049 | Inverted | - | 302 | NODE_4363-_length_304_cov_267.5:1-302 | NODE_917+_length_266619_cov_153.8:239789-240090 | 0 | 290 | 7 | 0 | 290 | 7 | 297 | 2.36 | Weak | 1.000 |
| 53 | Ralp_DISP_000050 | Inverted | - | 302 | NODE_4363-_length_304_cov_267.5:1-302 | NODE_1052_1959_copy_975_4397_4539+_length_303065_cov_137.417:40448-40749 | 0 | 235 | 5 | 1 | 235 | 6 | 241 | 2.49 | Weak | 1.000 |
| 54 | Ralp_DISP_000037 | Inverted | - | 302 | NODE_4363+_length_304_cov_267.5:3-304 | NODE_1052_1959_copy_975_4397_4539-_length_303065_cov_137.417:262317-262618 | 0 | 237 | 1 | 5 | 237 | 6 | 243 | 2.47 | Weak | 1.000 |
| 55 | Ralp_DISP_000043 | Direct | + | 302 | NODE_4363+_length_304_cov_267.5:3-304 | NODE_1052_1959_copy_975_4397_4539+_length_303065_cov_137.417:40448-40749 | 0 | 237 | 0 | 6 | 237 | 6 | 243 | 2.47 | Weak | 1.000 |
| 56 | Ralp_DISP_000092 | Direct | + | 280 | NODE_4466_4414_4515-_length_282_cov_577.31:1-280 | NODE_1052_1959_copy_975_4397_4539+_length_303065_cov_137.417:40497-40776 | 0 | 127 | 1 | 2 | 127 | 3 | 130 | 2.31 | Weak | 1.000 |
| 57 | Ralp_DISP_000048 | Direct | + | 302 | NODE_4363-_length_304_cov_267.5:1-302 | NODE_917-_length_266619_cov_153.8:26530-26831 | 0 | 284 | 3 | 0 | 284 | 3 | 287 | 1.05 | Weak | 1.000 |
| 58 | Ralp_DISP_000079 | Direct | + | 280 | NODE_4466_4414_4515+_length_282_cov_577.31:3-282 | NODE_1052_1959_copy_975_4397_4539-_length_303065_cov_137.417:262290-262569 | 0 | 129 | 0 | 2 | 129 | 2 | 131 | 1.53 | Weak | 1.000 |
| 59 | Ralp_DISP_000089 | Inverted | - | 280 | NODE_4466_4414_4515-_length_282_cov_577.31:1-280 | NODE_1052_1959_copy_975_4397_4539-_length_303065_cov_137.417:262290-262569 | 0 | 135 | 2 | 0 | 135 | 2 | 137 | 1.46 | Weak | 1.000 |
| 60 | Ralp_DISP_000076 | Inverted | - | 280 | NODE_4466_4414_4515+_length_282_cov_577.31:3-282 | NODE_1052_1959_copy_975_4397_4539+_length_303065_cov_137.417:40497-40776 | 0 | 142 | 0 | 2 | 142 | 2 | 144 | 1.39 | Weak | 1.000 |
| 61 | Ralp_DISP_000093 | Inverted | - | 280 | NODE_4466_4414_4515-_length_282_cov_577.31:1-280 | NODE_917-_length_266619_cov_153.8:26503-26782 | 0 | 145 | 2 | 0 | 145 | 2 | 147 | 1.36 | Weak | 1.000 |
| 62 | Ralp_DISP_000087 | Inverted | - | 253 | NODE_4466_4414_4515+_length_282_cov_577.31:30-282 | NODE_4363+_length_304_cov_267.5:52-304 | 0 | 0 | 1 | 0 | 0 | 1 | 1 | 100.00 | Very low | 1.000 |
| 63 | Ralp_DISP_000101 | Inverted | - | 253 | NODE_4466_4414_4515-_length_282_cov_577.31:1-253 | NODE_4363-_length_304_cov_267.5:1-253 | 0 | 0 | 0 | 1 | 0 | 1 | 1 | 100.00 | Very low | 1.000 |
| 64 | Ralp_DISP_000102 | Direct | + | 253 | NODE_4466_4414_4515-_length_282_cov_577.31:1-253 | NODE_4363+_length_304_cov_267.5:52-304 | 0 | 0 | 0 | 1 | 0 | 1 | 1 | 100.00 | Very low | 1.000 |
| 65 | Ralp_DISP_000075 | Inverted | - | 280 | NODE_4466_4414_4515+_length_282_cov_577.31:3-282 | NODE_917+_length_266619_cov_153.8:239838-240117 | 0 | 158 | 0 | 1 | 158 | 1 | 159 | 0.63 | Very low | 1.000 |
| 66 | Ralp_DISP_000080 | Direct | + | 280 | NODE_4466_4414_4515+_length_282_cov_577.31:3-282 | NODE_917-_length_266619_cov_153.8:26503-26782 | 0 | 159 | 0 | 1 | 159 | 1 | 160 | 0.62 | Very low | 1.000 |
| 67 | Ralp_DISP_000090 | Direct | + | 280 | NODE_4466_4414_4515-_length_282_cov_577.31:1-280 | NODE_917+_length_266619_cov_153.8:239838-240117 | 0 | 167 | 1 | 0 | 167 | 1 | 168 | 0.60 | Very low | 1.000 |

**Table S4. Summary of predicted mitochondrial RNA editing sites in *R. alpina* and R. purpurea.**

| table | Gene set | PCGs analyzed | Predicted editing sites | First codon position, n (%) | Second codon position, n (%) | Third codon position, n (%) | Synonymous, n (%) | Nonsynonymous, n (%) | Stop codons gained |
| --- | --- | --- | --- | --- | --- | --- | --- | --- | --- |
| *R. alpina* | All nonredundant PCGs | 36 | 403 | 138 (34.2%) | 235 (58.3%) | 30 (7.4%) | 36 (8.9%) | 367 (91.1%) | 3 |
| *R. purpurea* | All nonredundant PCGs | 34 | 390 | 143 (36.7%) | 219 (56.2%) | 28 (7.2%) | 33 (8.5%) | 357 (91.5%) | 4 |
| *R. alpina* | Shared PCGs | 32 | 354 | 130 (36.7%) | 199 (56.2%) | 25 (7.1%) | 31 (8.8%) | 323 (91.2%) | 3 |
| *R. purpurea* | Shared PCGs | 32 | 374 | 138 (36.9%) | 208 (55.6%) | 28 (7.5%) | 32 (8.6%) | 342 (91.4%) | 4 |
